# Applications of survival analysis and learning curves methods in neurosurgical stroke data and simulations to account for provider heterogeneity

**DOI:** 10.1186/s12874-025-02724-w

**Published:** 2025-12-09

**Authors:** Usha S. Govindarajulu, Rivera Daniel, Reynolds Eric, Brown Cole, Zhang Jack, Cohen Daniel, Schupper Alex

**Affiliations:** 1 Department of Population Health Science & Policy, Center for Biostatistics , Icahn School of Medicine at Mount Sinai, New York, NY 10029 USA; 2 Icahn School of Medicine at Mount Sinai, New York, NY 10029 USA; 3 Department of Neurosurgery, Icahn School of Medicine at Mount Sinai, New York, NY 10029 USA

**Keywords:** Survival analysis, Random survival forest, Learning curves, Restricted mean survival time, Neurosurgery

## Abstract

We used a unique application of Cox frailty models as well as random survival forests (RSF) to capture unexplained heterogeneity amongst providers (Int Neuro. Article 102149, 2025), along with using restricted mean survival time as an alternative survival time measure. Hemorrhagic stroke accounts for approximately 10–20% of all strokes annually and some patients with it may benefit from a surgical intervention, a placement of an external ventricular drain (EVD) catheter to manage post-procedure complications. In order to model post-surgical outcomes, we first employed frailty models or RSFs to capture heterogeneity between providers and account for unmeasured covariates (Int Neuro. Article 102149, 2025). Additionally, we had modeled learning curves among operators to guide and improve surgical learning in which we utilized our database of EVD procedures for hemorrhagic stroke interventions from 2019 to 2022, which revealed age was a significant predictor (*p* < 0.015) of time from procedure to death in an adjusted Cox model. In our novel modeling with frailty models along with restricted mean survival time, smoking became the main statistically significant predictor (Int Neuro. Article 102149, 2025), while RSFs showed the best fit in the real data as compared to the other methods. In this further exploration, simulations indicated that the exponential shape performed best while visually the logarithmic function performed best, aligning with prior research (Stat Med. 37:4185-4199, 2018), (Stat Med. 36:2764-2785PMC6463283, 2017), (J Med Stat Inform. 6, 2018).

## Introduction

External ventricular drainage (EVD) placement has long been one of the most frequently performed neurosurgical procedures. First described in 1744 by French surgeon Claude Nicholas Le Cat [4, 5], EVD has evolved into a critical, life-saving treatment for patients presenting with acute hydrocephalus, particularly in stroke cases involving intracerebral hemorrhage (ICH), subarachnoid hemorrhage (SAH), and intraventricular hemorrhage (IVH). The primary mechanism of EVD placement involves the drainage of cerebrospinal fluid (CSF), which alleviates pressure on surrounding brain tissue. This reduction in intracranial pressure (ICP) mitigates the risk of secondary brain injury and improves clinical outcomes. Additionally, EVD placement enables continuous ICP monitoring, providing crucial data to guide therapeutic decisions and clinical interventions [5, 6]. Despite its relative simplicity and frequent bedside performance, EVD placement carries inherent risks, including infections (e.g., ventriculitis), tract hemorrhage, and catheter obstruction necessitating readjustment or replacement[6, 7]. [8] Nevertheless, EVD remains an indispensable tool for neurosurgeons and neurocritical care physicians in managing patients with elevated ICP, particularly in stroke patients with ICH, SAH, and/or IVH.

In our previous work, we have modeled time to event and also incorporated this with the modeling learning curves [1, 3]. We wanted to extend this work to this EVD placement data, in which we had our own data collected on EVD procedures placed in adults greater than 18 years of age from 2019 to 2022 performed through the Mount Sinai Health System of New York City. The operators were physicians who were residents or faculty in the department of neurosurgery. The operators varied in experience and length of procedure, so it was of great interest to apply our learning curve methodology to this dataset. Through this retrospective chart review, we looked at time-to-event outcomes for these patients through standard survival techniques and also through our novel approach with frailty combined with restricted mean survival time (RMST) in order to account for potential heterogeneity between providers[2], through our own unique methods. We assessed both time to death as well as time to complication in separate analyses[2]. Furthermore, we also employed our learning curve analyses to better understand how case volume would affect procedural success and we employed different shapes for the learning curves, but this time we assessed these analyses in more detail through both extensive simulations as compared to the prior real data analyses. When simulating the data, we also tested how the number of surgeons and the censoring rate effected the learning curve model along with the different shape functions. We also used an array of different modeling methods along with shape functions to determine the optimal method of modeling the learning curve.

## Methods

We first conducted summary statistics of the variables collected where we obtained the frequencies of number of operators/physicians, number of cases/operators, prior history of stroke and CVD, patient gender, diabetes status, Modified Rankin Score (MRS), and complications. We also assessed the means/standard deviations, median, min and max values of patient age, survival time (from procedure date to death or last follow up and survival time from time of procedure to time to complication. We also assessed the complication and death rates and obtained proportions of these rates [1].

For time to event analyses, we analyzed time to event as time of procedure to either time to first complication post procedure or time to death [1]. We also employed right censoring for those who did not have the event. We first conducted a non-parametric Kaplan-Meier (KM) analyses for both event times as well as a log-rank test to compare the survival curves to each other. We then used the standard Cox proportional hazard model after confirming the proportional hazards assumptions met to model time to event, either time to complication or time to death, and adjusted by EVD type, age, gender, anti-coagulant use, history of stroke, history of diabetes (yes or not), history of CVD, history of drug use, history of hypertension, history of smoking, and ICH or IVH or SAH diagnoses as well as clustering by physician to account for potential correlations within each physician’s cases[1].

In a novel application for this dataset, we were also interested in a frailty analysis to discern heterogeneity between operators and conducted a Cox frailty model analysis [1], which we had done previously but explain this in more mathematical detail. Also, in this development and in more mathematical detail, we also used RMST [8, 9] to estimate survival probabilities instead of a Cox model or machine learning method, adjusted by covariates, along with incorporating frailty in a new development, to handle unexplained heterogeneity. RMST has been used as an alternative metric for summarizing survival time distributions, where it is the mean survival over all subjects in the study but only up to a particular time point, *t*^89^, which is denoted as $\:\tau\:$. If the observed data is not correlated, a natural nonparametric estimator for $\:RMS{T}_{k}\left(\tau\:\right)$ where k was the number of groups was given by plugging the Kaplan-Meier estimator, $\:{\widehat{S}}_{k}\left(t\right),$ for $\:{S}_{k}\left(t\right),$

1 $$\:{\widehat{RMST}}_{k}\left(\tau\:\right)={\int\:}_{0}^{\tau\:}{\widehat{S}}_{k}\left(u\right)du.$$

For correlated data, instead of $\:{\widehat{S}}_{k}\left(u\right),\:$we used the baseline survival function estimated from a stratified Cox regression model with shared frailty with grouping by operators. Specifically, we consider the following frailty model,

2 $$\Lambda_{ij}\left(t\mid w_j,Z_{ij},X_{ij}\right)=w_j{\Lambda\:}_{Z_{ij}}\left(t\right)\text{exp}{\beta\:}^{'\:}X_{ij},$$

where $\:{{\Lambda\:}}_{ij}(\cdot\:)$ is the cumulative hazard function for the $\:i$^th^ subject in the $\:j$^th^ cluster, $\:{Z}_{ij}$ is a variable to determine the stratum (e.g., the assigned treatment group indicator), $\:{w}_{j}$ is the frailty term with mean 1 from a gamma distribution and variance of *θ*, and $\:{X}_{ij}$ is the covariate vector for adjustment.

We then again our learning curve (LC) methods [1, 2] to adjust the time to death analyses by infusing a particular form of a parametric LC shape (exponential, power series, logarithmic, and log-normal), and then ran the models with an ordered procedure variable per operator to model learning within the adjusted Cox model. We obtained the predicted survival from the stratified Cox model.

3 $$h_{ij}\left(t\right)=h_{0s}\left(t\right)exp\left(x_{ij}\beta+w_i\right)\\{\widehat p}_i={\widehat S}_i\left(t\right)={\widehat S}_0\left(t\right)exp\left(\widehat\beta x_i\right)$$

where *h*_*0s*_*(t)* denotes the stratified baseline hazard rate. The individual level estimated survival probabilities from the Cox model were denoted as *p*_*i*_ for each observation as computed from multiplying the baseline survival rate, $\:\widehat{{S}_{0}}\left(t\right)$, by the function of the covariates, $\:\text{e}\text{x}\text{p}\left(\widehat{\beta\:}{x}_{i}\right)$. The *p*_*0i*_ are the predicted probabilities derived from Eq. 1 as *p*_*i*_, which is the asymptotic steady state outcome event rate in the simulation model.

These survival times were adjusted or updated for the physician learning curve, p1, for each shape separately, and then new survival probabilities where calculated and plotted against procedural volume. The *p*_*1*_ are the predicted probabilities from the physician learning curve model for the particular shape which were used to then calculate *p*_*2*i_ for the center learning curve and these were the final predicted probabilities. The final predicted probabilities were re-constructed as an updated survival time, *tnew*_*i*_ = (*p*_*2i*_*/p*_*0i*_)*time_*i*_ by taking a ratio of the final probabilities, *p*_*2i*_, by the asymptotic steady state probabilities, *p*_*0i*_. This updated survival time became the new time variable in the previous dataset to generate new success probabilities. We also employed this in a Cox frailty model and the random survival forest (RSF) [9, 10] instead of just the Cox model to estimate survival in order to model learning curves between survival probabilities and procedure volume with these other methods. In our new development, we also employed the RMST in our learning curve framework and then compared how well using this compared to the clustered or frailty Cox model and the RSF.

To evaluate the robustness of our learning curve models, we conducted extensive simulation studies which was based on our prior published methodology [1, 2, 11, 12] and adapted for this neurosurgical data using the same covariates as from the real data analysis with similar distributional assumptions for the parameters. These simulations assessed the impact of various factors on model performance, including the number of surgeon operators (5 or 10 per center) and censoring rates (10% & 70%). We also tested four different shape functions (exponential, power series, logarithmic, log-normal) to determine their effectiveness in capturing learning patterns under various conditions. Through these simulations, we aimed to identify the optimal methods and shape functions for modeling surgical learning curves across a range of scenarios.

For each method and shape we show plots where each observed point in these figures is the binned average of predicted probabilities of success for the particular shape and scenario along with the corresponding learning curve. We also calculated root mean square error (rMSE) between the predictions and the marginal success rates in the observed data using our prior methods for this [1, 2] to see if these results aligned with the graphical results. All simulations and analyses were conducted in the R software. We used the libraries, survival, frailtypack, and our own R program for the RMST frailty analyses. We used a significance level of 0.05 for all analyses.

## Results: survival analyses on real data

### Univariate analyses

The study population was created from 652 patient medical records between 2019 and 2022. After removing duplicate subjects and subjects who did not meet eligibility criteria or subjects that had confidential records and could not be included we had 439 subjects. Out of these 439 patients after removing patients missing key information the final study population consisted of 160 patients. Our Table 1 had laid out clear demographics amongst the study population [1]. Our study population consisted of patients over the age of 18 who had an EVD procedure done after having an SAH, ICH, or IVH event. These patients were treated with either Bactericidal, IRRAflow or Cerebroflo© EVD devices and were not missing any key variables. For analytical purposes, we combined the IRRAflow and Cerebroflo devices and called that combination the non-antibiotic EVD devices as compared to the Bactericidal antibiotic device. The median age in the study population was 59 with the minimum age being 27 years old and the maximum patient age was 93. Regarding the patient diagnosis, we observed that 53% (84) had SAH, 41% (65) had IVH and 47% (75) had ICH diagnoses. Amongst the 429 subjects not included in the final study population, EVD type (~ 40%) and History of Smoking (~ 22%) had the most missing or unknown observations. In most cases data was not missing but rather results could not be assigned to a specified category resulting in them being treated as unknown for the sake of analysis. Approximately 64% (103/160) were treated with Bactericidal devices, the other 36% (57/160) were treated with IRRAflow/Cerebroflo©. The racial composition was diverse. Approximately 26% (42) of the subjects were Black or African-American, 24% (38) were Hispanic, 21% (33) were White, 11% (17) were Asian and 19% had a races that was Unknown or missing. Meanwhile the gender distribution was nearly equal with approximately 51% (81) were male and 49% (78) were female. Approximately 28% (44) of patients received anti-coagulants during treatment. Post-procedure mortality was approximately 26% (41) of patients while 17% (27) of patients experienced a complication post procedure.

**Table 1:** .

Variable	Overall (*N* = 160) Mean (sd) or *n* (%)
**Age**	57.5 (14.5)
**Gender**	
Male	81 (50.6%)
Female	78 (48.8%)
**Center**	
EHC	11 (6.9%)
MSH	91 (56.9%)
MSM	8 (5.0%)
MSW	48 (30.0%)
**Race**	
Asian	17 (10.6%)
Black	42 (26.3%)
Hispanic	38 (23.8%)
White	33 (20.6%)
Unknown	30 (18.8%)
**Height**	65.7 (4.05)
**Weight**	175 (40.7)
**BMI**	29.2 (12.2)
**Diabetes**	20 (12.5%)
**History Stroke**	18 (11.3%)
**History Hypertension**	55 (34.4%)
**Anti-coagulant Use**	44 (27.5%)
**Smoke**	78 (48.8%)
**History CVD**	24 (15.0%)
**Pre-OP MRS**	2.93 (1.67)
**SAH**	84 (52.5%)
**IVH**	65 (40.6%)
**ICH**	75 (46.9%)
**Device**	
IRRAflow/Cerebroflo©	57 (35.6%)
Bactericidal	103 (64.4%)
**Time to Death**	31.8 (30.4)
**Status - Death**	41 (25.6%)
**Time to Complication**	27.2 (29.9)
**Status - Complication**	27 (16.9%)

### Time to death analyses

**Fig. 1:**
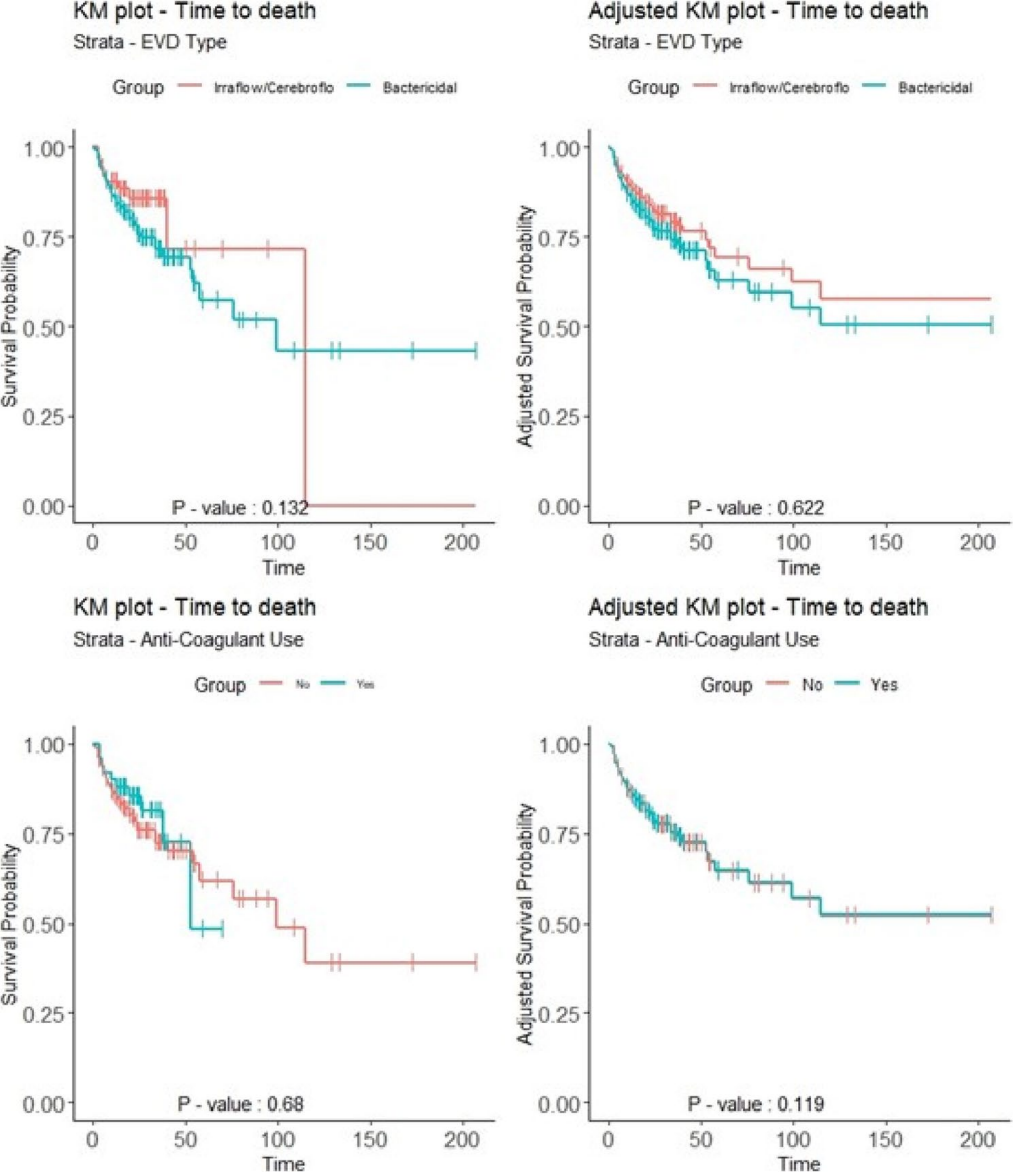
Stratified KM plots: time to death stratified by EVD type or anticoagulant use

Kaplan-Meier plots for time of procedure to time to death, both unadjusted as well as adjusted (Fig. 1) as per prior analyses [1], revealed that anti-coagulant use did not show a significant difference in survival initially. However, after adjusting for other covariates, at which point the survival rate for those on anti-coagulants appeared higher. Also, in terms of the same types of plots for EVD device we saw even after adjustment that there was not any statistically significant difference between the devices being compared, IRRAflow/Cerebroflo© and Bactericidal. We found in the Cox proportional hazards regression for time of procedure to time of death with physician clustering (Table 2) [1], that age was a significant risk factor in both univariate (*p* < 0.001) and multivariate (*p* < 0.015) models. However, pre-operative MRS, history of stroke, and history of smoking, which were initially statistically significant in the univariate models (P-values respectively: <0.006, < 0.027, < 0.006) became borderline or not statistically significant in the multivariable model (multivariable p-values respectively: <0.074, < 0.10, < 0.13).

**Table 2:** Cox model for time to death with physician clustering

Characteristic	Unadjusted			Adjusted		
	HR^*1*^	95% CI^*1*^	p-value	HR^*1*^	95% CI ^*1*^	p-value
Age	1.03	1.01, 1.06	0.001	1.04	1.01, 1.07	0.015
Gender	0.83	0.48, 1.43	0.5	1.23	0.60, 2.52	0.6
Diabetes	0.91	0.36, 2.29	0.8	0.55	0.15, 1.99	0.4
History Stroke	2.17	1.09, 4.33	0.027	2.22	0.87, 5.65	0.10
History Hypertension	1.45	0.84, 2.52	0.2	0.93	0.37, 2.39	0.9
Anticoagulant use	0.64	0.34, 1.22	0.2	0.45	0.18, 1.12	0.087
History Smoke	2.52	1.31, 4.86	0.006	1.83	0.84, 4.02	0.13
SAH Diagnosis	1.19	0.69, 2.04	0.5	2.25	0.59, 8.53	0.2
IVH Diagnosis	0.92	0.54, 1.59	0.8	1.10	0.51, 2.35	0.8
ICH Diagnosis	1.00	0.58, 1.71	> 0.9	2.39	0.68, 8.43	0.2
Pre-OP MRS	1.31	1.08, 1.59	0.006	1.24	0.98, 1.56	0.074
History CVD	1.73	0.92, 3.26	0.089	1.01	0.43, 2.39	> 0.9
History Drug Use	0.82	0.40, 1.68	0.6	0.74	0.29, 1.90	0.5
EVD Type^2^	1.26	0.70, 2.29	0.4	1.11	0.47, 2.62	0.8

^1^ *HR* Hazard Ratio, *CI* Confidence Interval

^2^Reference value: IRRAflow/Cerebroflo©

#### Time to complication analyses

**Fig. 2:**
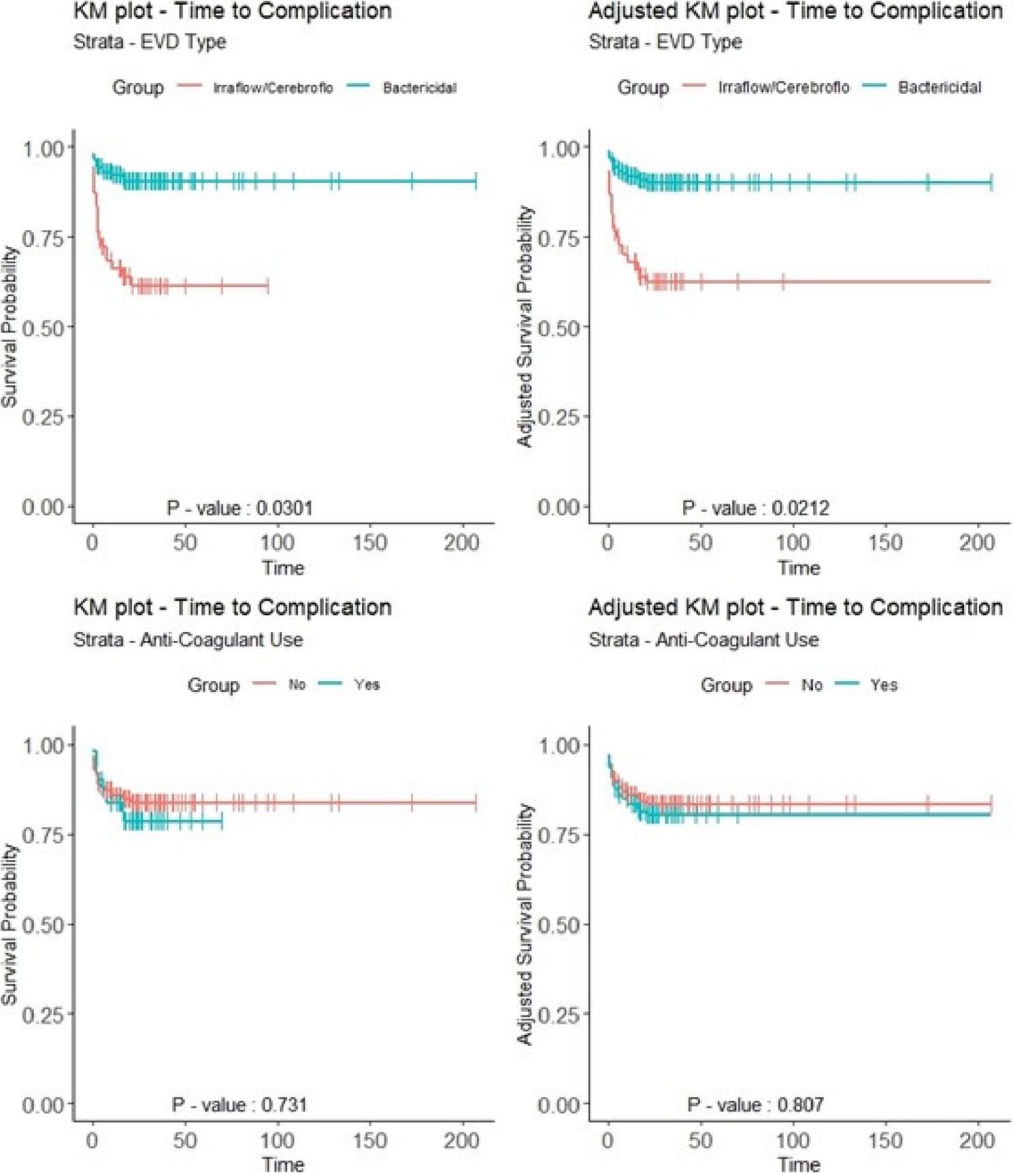
Stratified KM plots: time to complication stratified by EVD type of anticoagulant use

For the Kaplan-Meier plots for time of procedure to time of first complication (Fig. 2), we found no survival difference in anti-coagulant use between unadjusted and adjusted analyses as per prior analyses [1], however, we did find a survival difference between the EVD device types with Bactericidal showing a higher probability of survival on average, but unadjusted and adjusted analyses showed similar rates of survival between devices.

For the Cox proportional hazard regression model for time to complication (Table 3) as per prior analyses [1], we found that EVD device type remained as a significant predictor in either univariate (*p* < 0.008) or multivariate (*p* < 0.010) Cox model analyses while pre-operative MRS was initially significant in the univariate analysis (*p* < 0.014) but became borderline significant in the multivariate analysis (*p* < 0.060).

**Table 3:** Cox model for time to complication with physician clustering

**Characteristic**	Simple			Adjusted		
	**HR** ^*1*^	**95% CI** ^*1*^	**p-value**	**HR** ^*1*^	**95% CI** ^*1*^	**p-value**
Age	1.00	0.97, 1.03	> 0.9	1.00	0.97, 1.04	0.8
Gender	0.86	0.41, 1.82	0.7	1.04	0.44, 2.44	> 0.9
Diabetes	0.63	0.15, 2.66	0.5	0.47	0.09, 2.35	0.4
History Stroke	1.12	0.34, 3.72	0.9	0.75	0.19, 2.93	0.7
History Hypertension	1.61	0.76, 3.45	0.2	1.29	0.41, 4.07	0.7
Anticoagulant use	0.98	0.43, 2.22	> 0.9	0.81	0.31, 2.09	0.7
History Smoke	0.99	0.46, 2.10	> 0.9	0.66	0.25, 1.77	0.4
SAH Diagnosis	0.90	0.43, 1.88	0.8	0.96	0.26, 3.54	> 0.9
IVH Diagnosis	1.54	0.73, 3.23	0.3	1.94	0.75, 4.97	0.2
ICH Diagnosis	0.67	0.31, 1.43	0.3	0.60	0.19, 1.92	0.4
Pre-OP MRS	0.75	0.60, 0.94	0.014	0.79	0.62, 1.01	0.060
History CVD	2.26	0.96, 5.32	0.062	2.30	0.64, 8.35	0.2
History Drug Use	0.49	0.21, 1.16	0.11	0.45	0.14, 1.38	0.2
EVD Type^2^	0.36	0.17, 0.77	0.008	0.35	0.15, 0.78	0.010

^1^ *HR * Hazard Ratio, *CI* Confidence Interval

^2^Reference value : IRRAflow/Cerebroflo©

### Frailty analyses

As per prior analyses [1], in the regular Cox frailty model, none of the variables showed a statistically significant effects on time to death (Table 4), with history of smoking variable (*p* < 0.0640) being the closest to significance. In the RMST Cox frailty model, history of smoking had an even more statistically significant effect in the model (*p* < 0.0534) as compared to the Cox frailty model. The frailty p-value was not statistically significant in either the regular Cox frailty or RMST Cox frailty models, which indicated that there was no significant heterogeneity in care between different providers. These analyses contradicted the regular Cox model analyses for time to death with physician clustering which had identified age as the main significant predictor so clearly, taking into account the provider heterogeneity altered the results for the main predictor of mortality.

**Table 4:** Cox frailty & RMST frailty models: real data analyses

Variable	Cox frailty model				RMST Cox frailty			
	coef	Se(coef)	P-value	Frailty p-value	coef	Se(coef)	P-value	Frailty
Age	0.010	0.0152	0.5200	0.95	0.010	0.0152	0.5004	Var: 4.13 x e^− 15^ *P* = 0.5
Gender	0.174	0.3744	0.6400		0.219	0.3767	0.5607	
Diabetes	−0.123	0.6355	0.8500		−0.143	0.6098	0.8148	
Pre-op MRS	0.153	0.1287	0.2300		0.157	0.1287	0.2221	
History Stroke	0.380	0.5418	0.4800		0.409	0.5587	0.4647	
History Hypertension	0.027	0.5311	0.9600		−0.003	0.5370	0.9951	
Anti-coagulant Use	−0.412	0.4866	0.4000		−0.415	0.4848	0.3918	
Smoke	0.712	0.3846	0.0640		0.739	0.3825	0.0534	
SAH	0.316	0.5942	0.5900		0.267	0.6000	0.6568	
IVH	0.275	0.3917	0.4800		0.301	0.3982	0.4500	
ICH	0.481	0.5569	0.3900		0.455	0.5604	0.4164	
History CVD	0.450	0.4811	0.3500		0.433	0.4719	0.3588	
History Drug Use	0.511	0.3794	0.1800		0.508	0.3730	0.1728	
Device	0.262	0.2293	0.2500		NA			
					RMST DIFFERENCE	13.48 (21.1)	95% CI: (27.86, 54.83)	

## Results: learning curve real data analysis

**Fig. 3:**
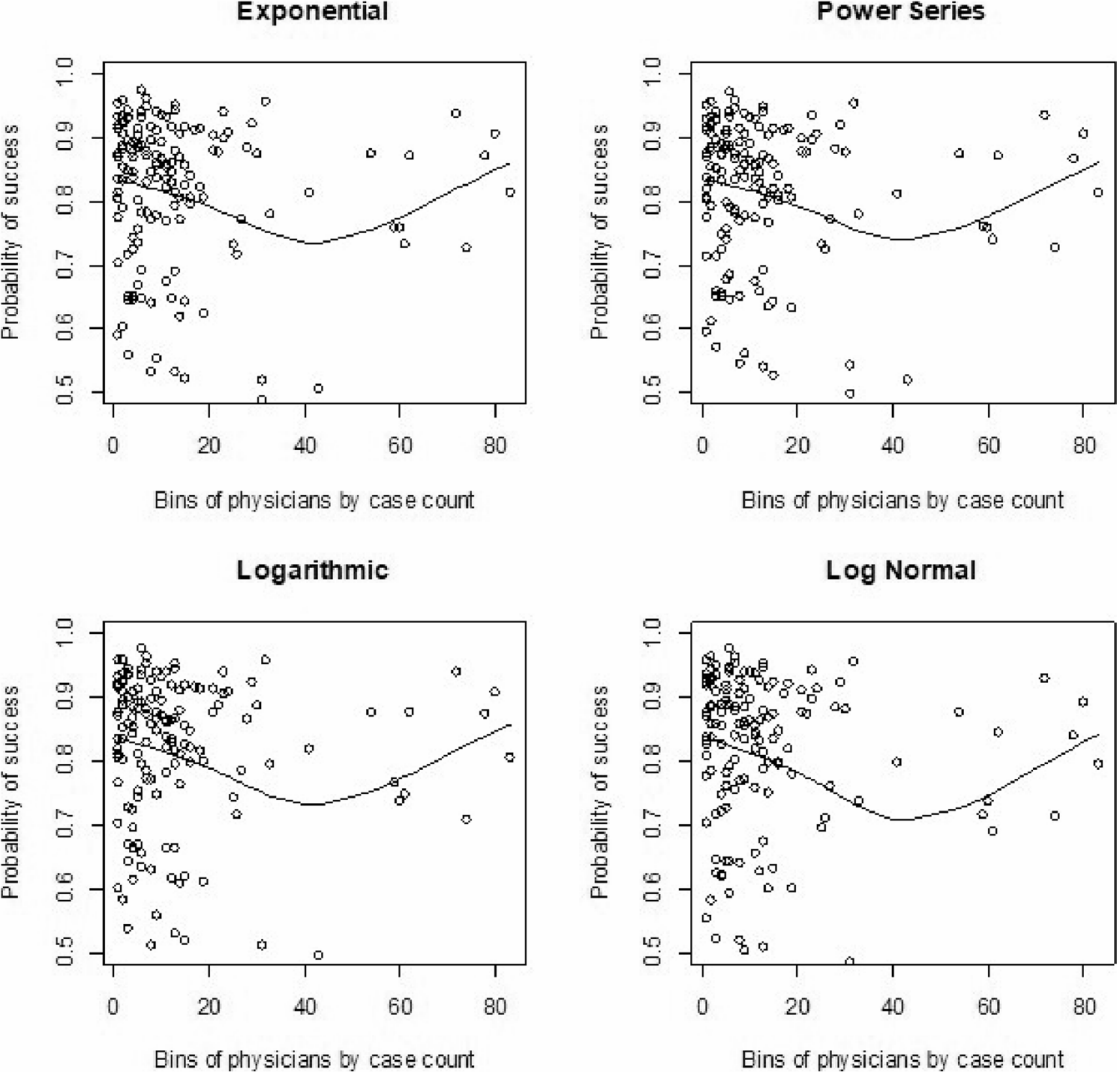
Cox model: real data analysis learning curve plots for 4 shapes (exponential, power series, logarithmic, and log-normal) and observed average points

**Fig. 4:**
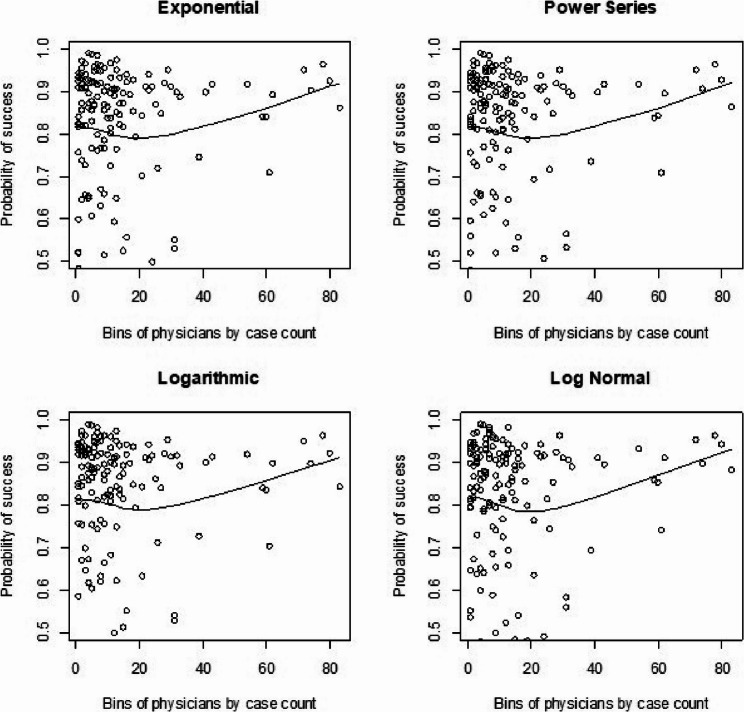
Cox frailty model: real data analysis learning curve plots for 4 shapes (exponential, power series, logarithmic, and log-normal) and observed average points

**Fig. 5:**
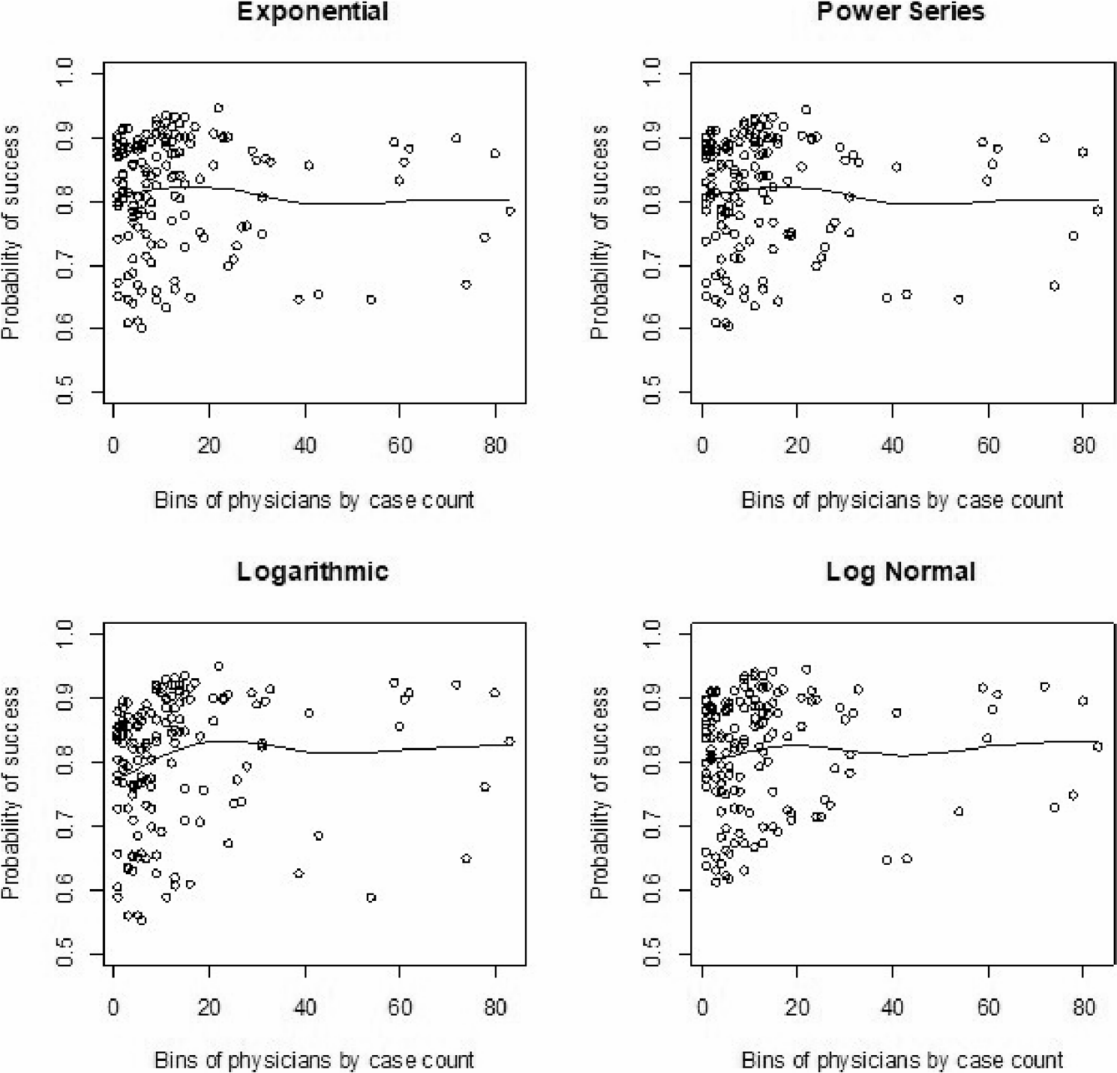
Random survival forest: real data analysis learning curve plots for 4 shapes (exponential, power series, logarithmic, and log-normal) and observed average points

We had also shown visual representations for learning curves of the probability of procedural success by plotting our predicted survival probabilities from each model against procedural volume from our real data for time to death analyses. We show this here again [1] for comparison to the simulated learning curves. For the standard Cox model with clustering by physicians (Fig. 3) we saw a consistent dip across shapes around 40 cases and then back to an increase after that in the learning curve. The Cox frailty plots (Fig. 4) showed a more consistent and upward learning curve across case volume, aligning with expectations of continuous improvement. However, the RSF learning curve plots (Fig. 5) we did not show much of any learning trend. Finally, for the mean-squared error calculations (Table 5) between observed and fitted values, we found that the RSF seemed to fit the trajectories the closest while the Cox frailty model that showed an expected learning curve trajectory was farthest from the observed data points. A potential issue with that was that the majority of observations were clustered in the lower-case order.AQ

**Table 5:** Learning curve Models – Mean squared error

	Model		
Shape	Regular Cox	Frailty Cox	Random Survival Forest
Exponential	0.1176	0.1692	0.0883
Power Series	0.1138	0.1693	0.0878
Logarithmic	0.1243	0.1718	0.0988
Log-Normal	0.1303	0.1706	0.0890

The mean squared error above compare the observed with the predicted estimates from the model

## Simulation learning curve results

We conducted learning curve simulations for the regular Cox model, the regular Cox frailty model, RMST Cox frailty, and the RSF across sixteen scenarios to assess the best fitting method. For the simulation framework, we varied the number of surgeons, the censoring rate, and the assumed shape (exponential, power series, logarithmic, or log-normal) across these scenarios (Table 6). For all the models, we performed 50 simulations per scenario. For the RMST frailty model, we were only able to perform 10 simulations per scenario due to computational constraints.

**Table 6:** Simulation scenarios

Scenario	Number of physicians/center	Censoring	Shape
1	10	10%	Exponential
2	10	10%	Power Series
3	10	10%	Logarithmic
4	10	10%	Log-Normal
5	5	10%	Exponential
6	5	10%	Power Series
7	5	10%	Logarithmic
8	5	10%	Log-Normal
9	10	70%	Exponential
10	10	70%	Power Series
11	10	70%	Logarithmic
12	10	70%	Log-Normal
13	5	70%	Exponential
14	5	70%	Power Series
15	5	70%	Logarithmic
16	5	70%	Log-Normal

**Fig. 6:**
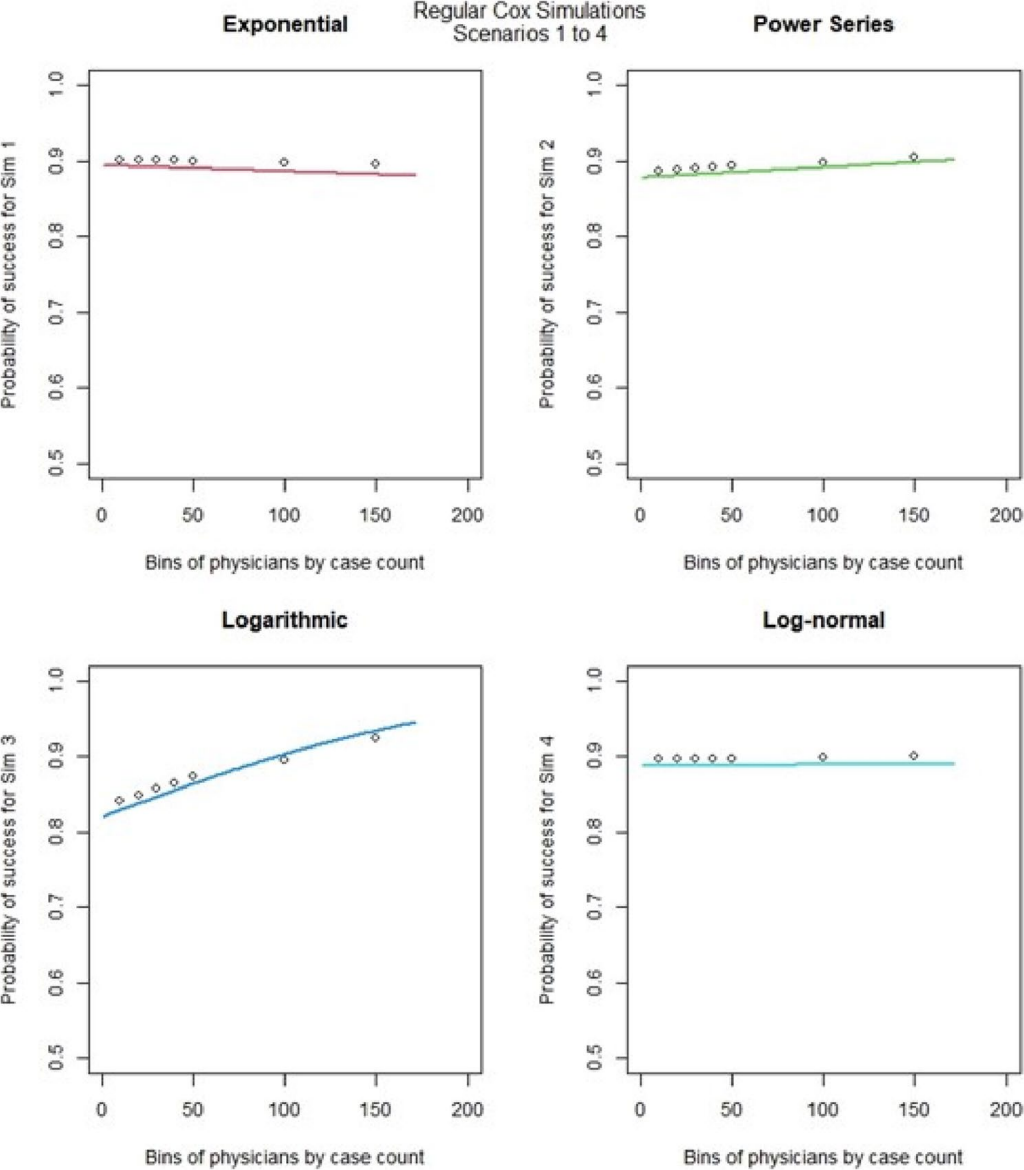
Cox model : simulations 1-4 learning curve plots for 4 shapes (exponential, power series, logarithmic, and log-normal) and observed average points

**Fig. 7:**
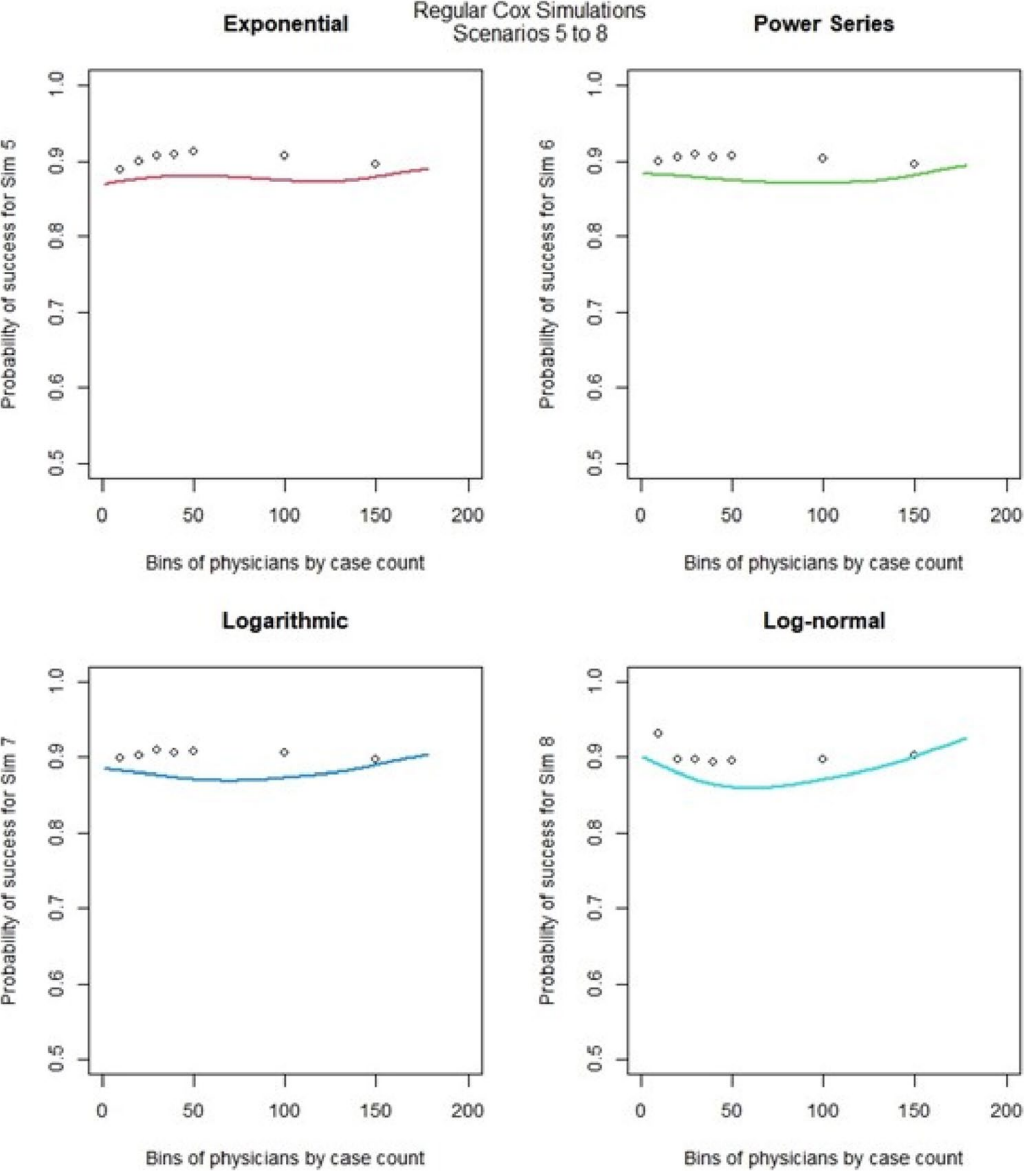
Cox model : simulations 5-8 learning curve plots for 4 shapes (exponential, power series, logarithmic, and log-normal)and observed average points

**Fig. 8:**
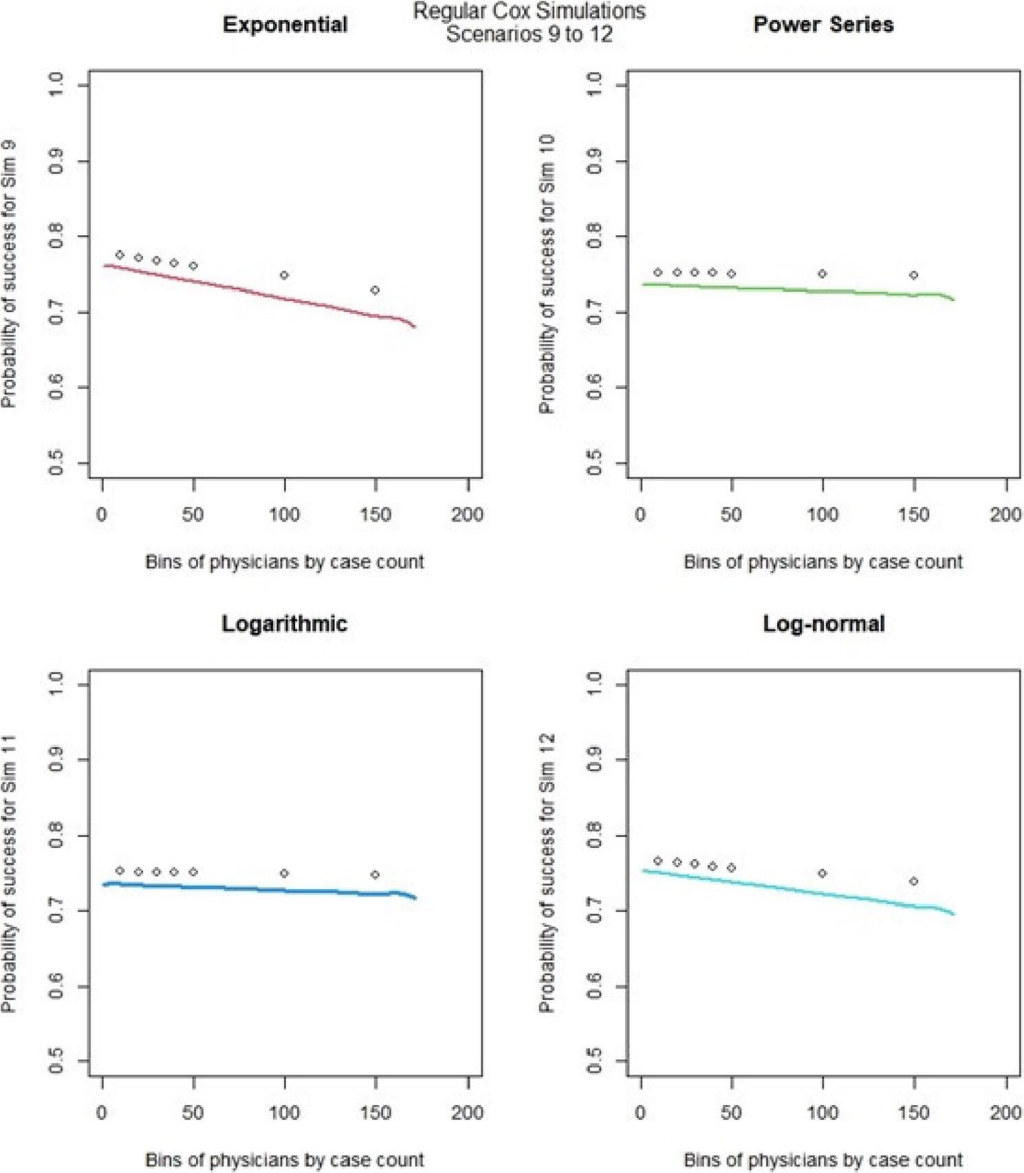
Cox model : simulations 9-12 learning curve plots for 4 shapes (exponential, power series, logarithmic, and log-normal)and observed average points

**Fig. 9:**
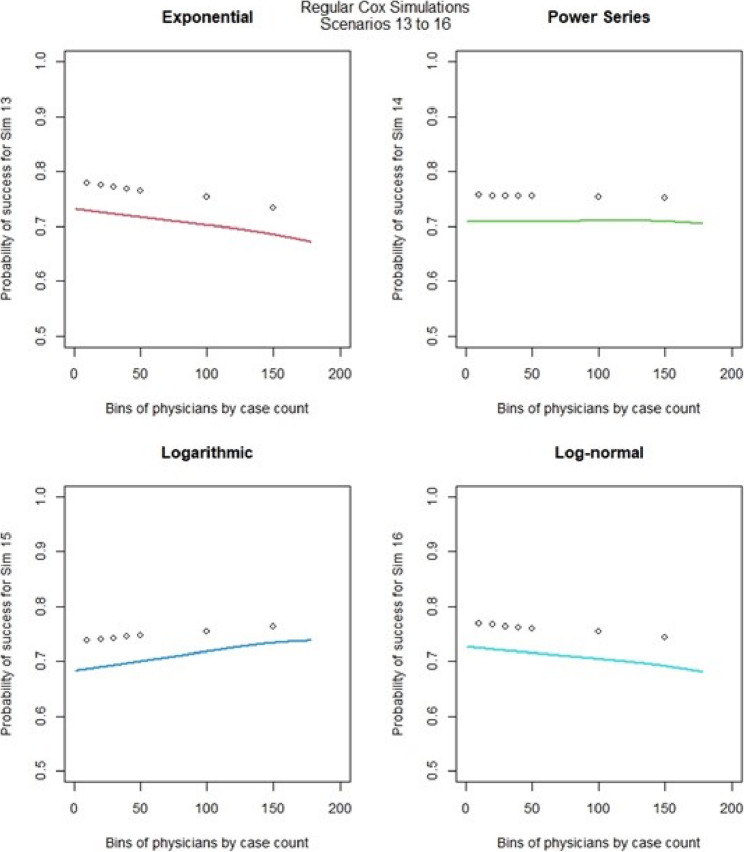
Cox model : simulations 13-16 learning curve plots for 4 shapes (exponential, power series, logarithmic, and log-normal) and observed average points

For the regular Cox models, scenarios 1 to 4, which had more surgeons and low censoring rate (Fig. 6), we found that the logarithmic shape had the best graphical fit. While for scenarios 5 to 8 (Fig. 7), which had fewer surgeons and low censoring rate this was similar to scenarios 1 to 4, were we saw that the logarithmic model seemed to have the best fit. Scenarios 9 to 12 (Fig. 8), which had higher censoring rate and more surgeons did not show any of the shapes as well fitting to the learning curve. Scenarios 13 to 16 (Fig. 9), which had higher censoring and less surgeons, showed that the logarithmic shape fitted the best, but it was not as well fitting as in other scenarios. Across the 16 scenarios we saw that the logarithmic shape provided the best results for the regular Cox model while we found that increasing censoring reduced the accuracy of the model and having more surgeons led to more accurate results. This aligned with our previous learning curve research [1–4].

**Fig. 10:**
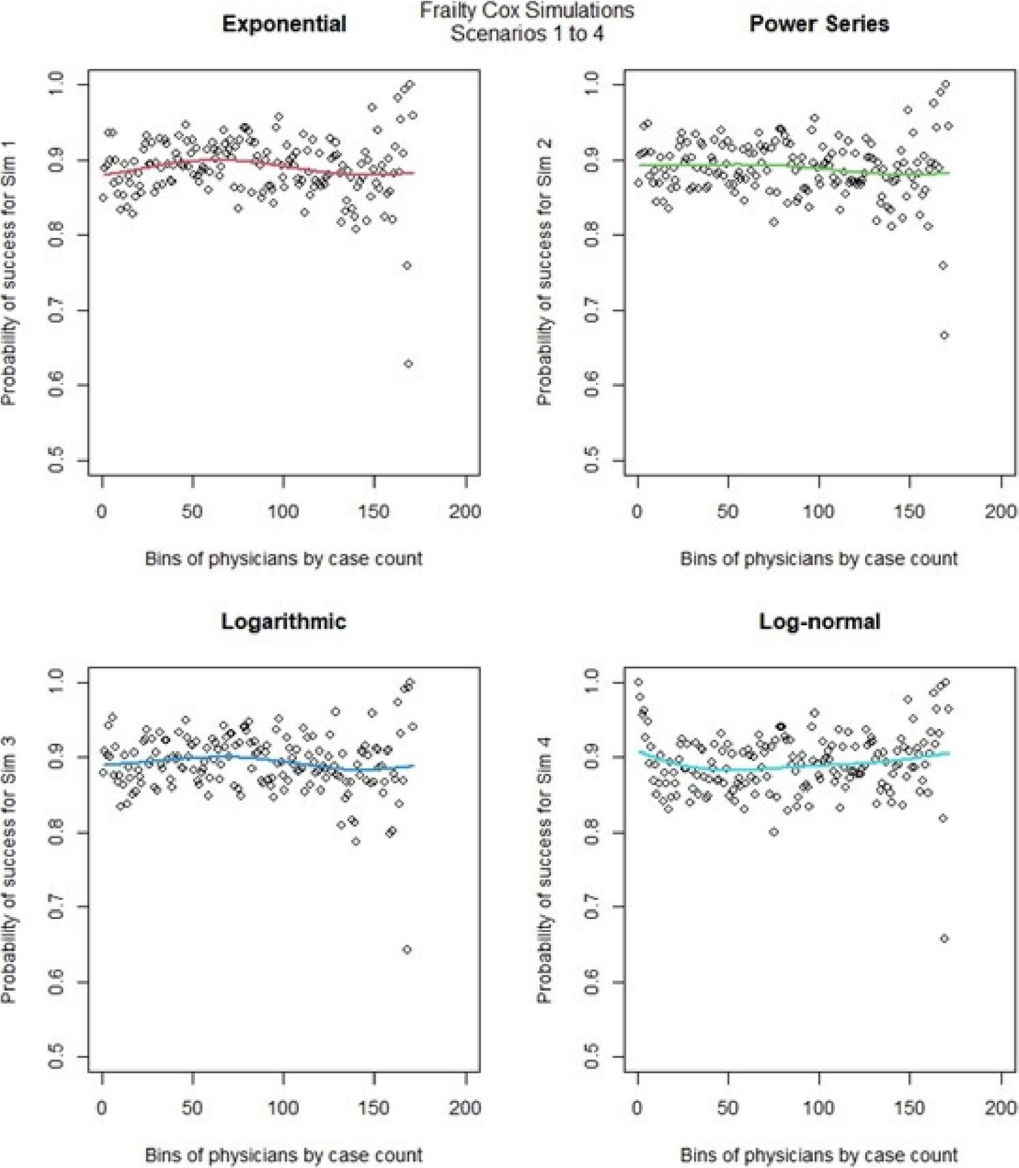
Cox frailty model : simulations 1-4 learning curve plots for 4 shapes (exponential, power series, logarithmic, and log-normal) and observed average points

**Fig. 11:**
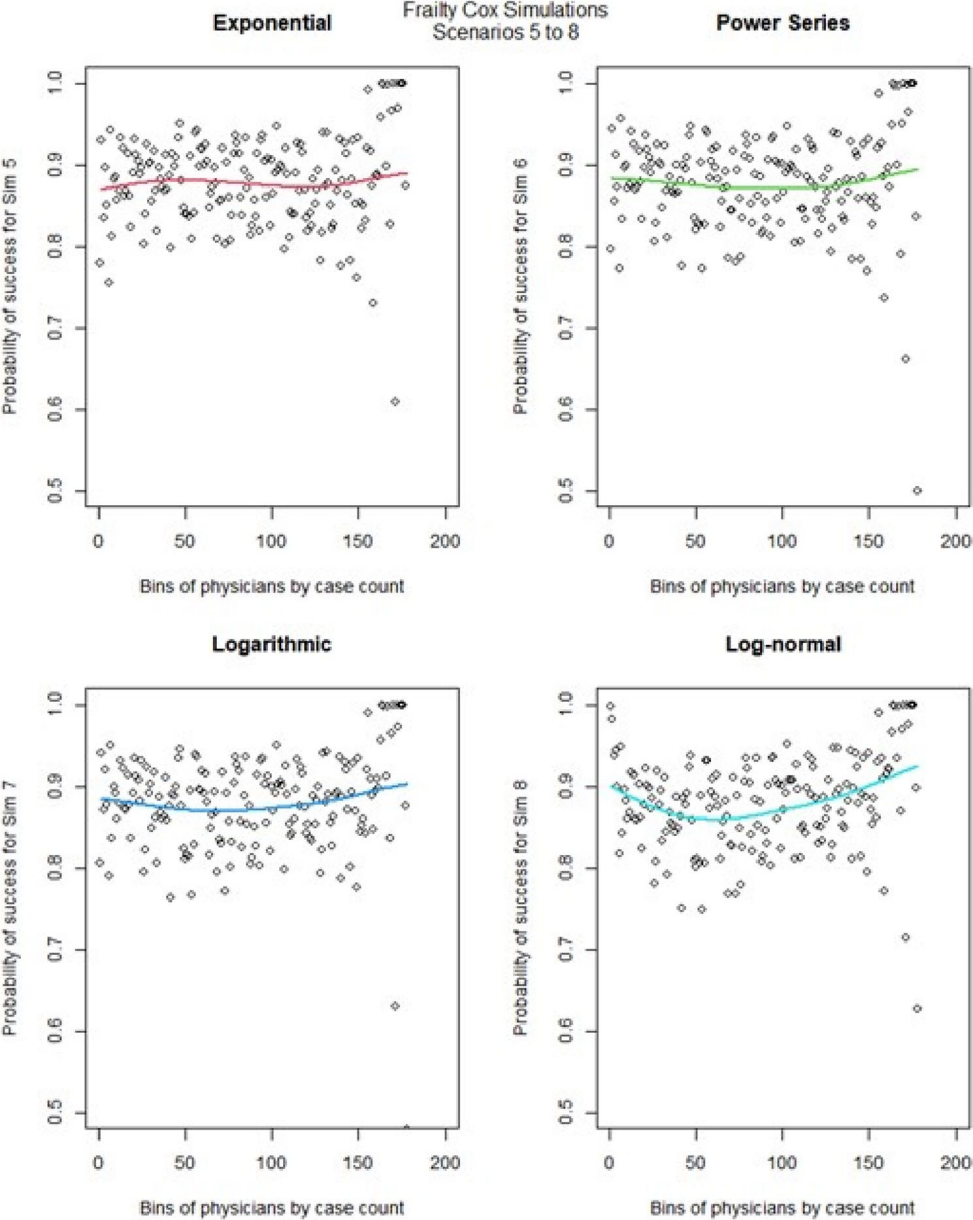
Cox frailty model : simulations 5-8 learning curve plots for 4 shapes (exponential, power series, logarithmic, and log-normal) and observed average points

**Fig. 12:**
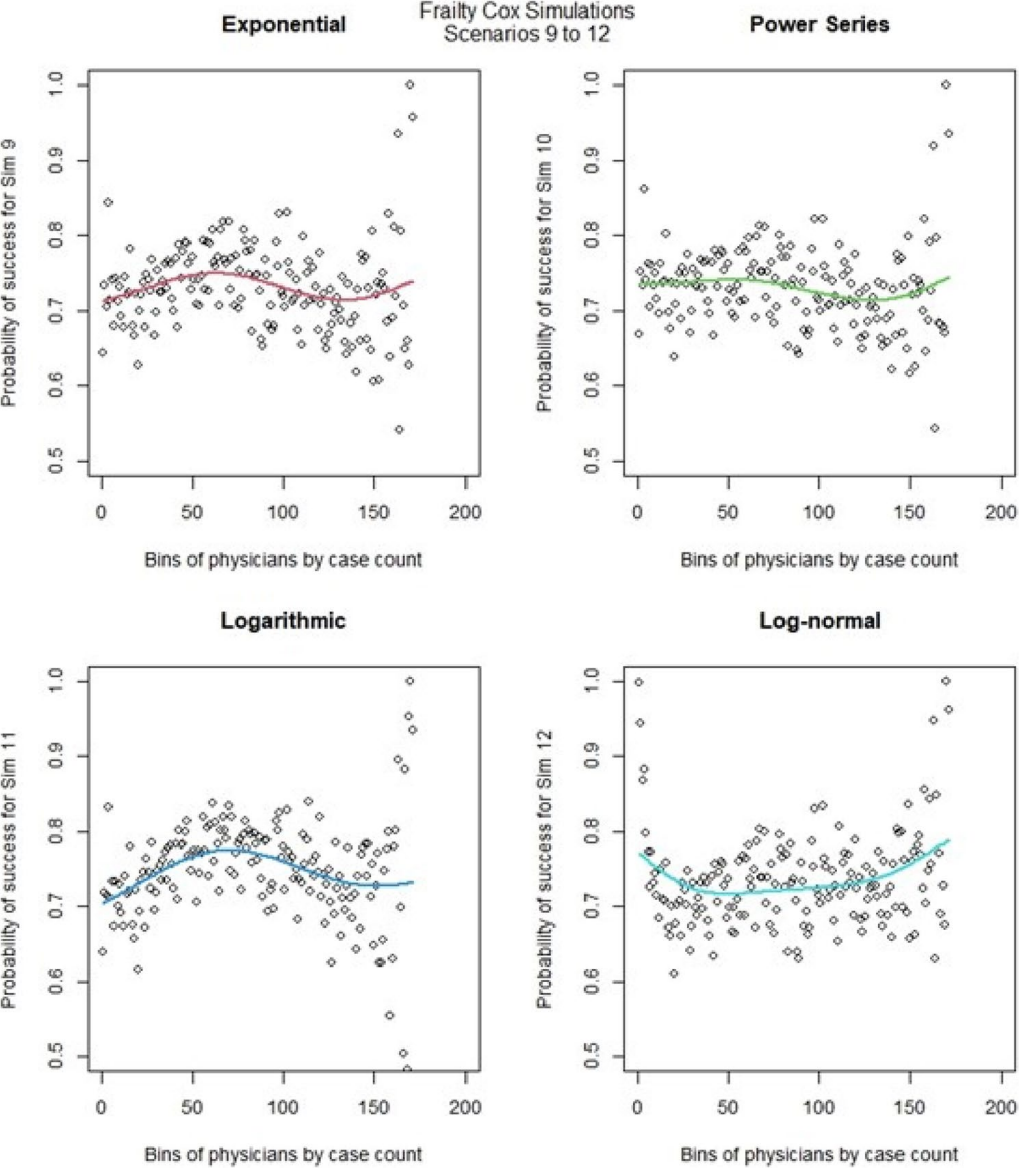
Cox frailty model : simulations 9-12 learning curve plots for 4 shapes (exponential, power series, logarithmic, and log-normal) and observed average points

**Fig. 13:**
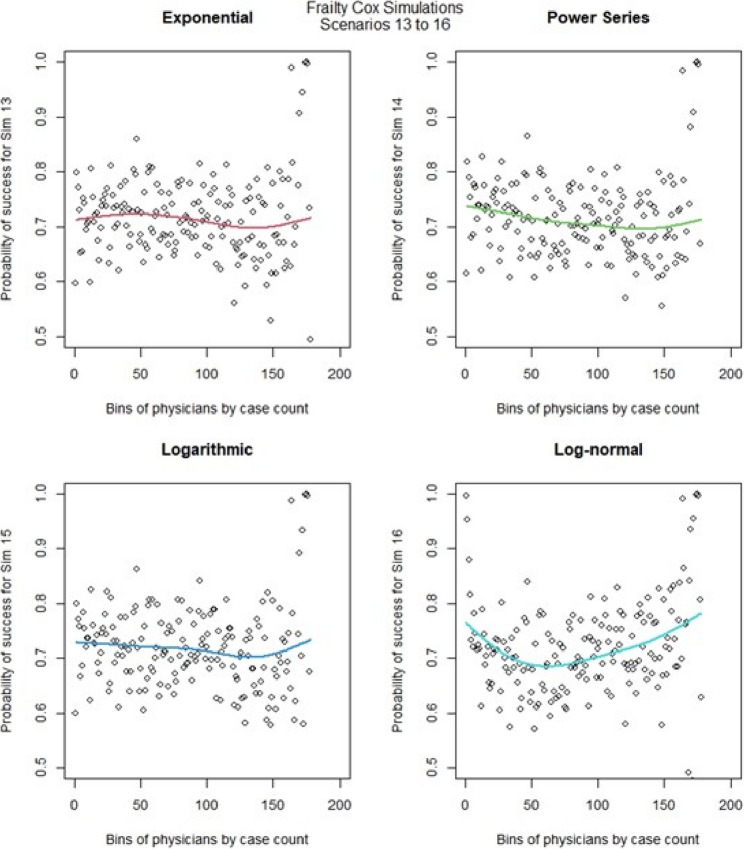
Cox frailty model : simulations 13-16 learning curve plots for 4 shapes (exponential, power series, logarithmic, and log-normal)

We then ran the Cox frailty model for the learning curve for all 16 scenarios. The most notable change came from altering the censoring rate. When censoring was low (Fig. 10, 11,) we observed that the exponential & logarithmic functions showed similar fits to the data. The log-normal results were the best if we consider its behavior after 50 cases. None of the shape functions used gave particularly accurate results. Scenarios 9 to 16 (Fig. 12, 13) had high censoring so the results were less predictable. The results were similar in most cases when censoring was high, the only exception is that the log-normal performed better when there were less surgeons in the model. The Cox frailty model showed inconsistent results. The MSEs from Table 7 would indicate the Cox frailty and the regular Cox model were fairly consistent across all scenarios between the fit between predicted survival and average survival and observed points. The logarithmic shape also in general showed lower MSEs across the scenarios as compared to the other shapes. For more granularity for the RSF and the Cox RMST frailty, sometimes the RSF was better than the Cox RMST frailty at fitting the actual shape as compared to p0 (baseline) predictions and sometimes worse, but the Cox RMST frailty did better with the fit between both p1 (physician) and p2 (center) predictions as compared to p0 than the RSF fits to the same type of predictions. For the RSF, the exponential and power series performed better across the scenarios in terms of MSEs while for the Cox RMST frailty, the logarithmic still seemed to perform better though we could not ascertain its fit in scenario 3 due to lack of convergence.

**Table 7:** – Simulations: mean squared error

Model	Regular Cox	Frailty Cox	Random Survival Forest			RMST Cox frailty		
Scenario	MSE1	MSE1	MSE2	MSE2_a	MSE2_b	MSE2	MSE2_a	MSE2_b
1	0.0101	0.0104	0.0192	0.0041	0.0054	0.0354	0.0089	0.0050
2	0.0079	0.0106	0.0479	0.0062	0.0052	0.0512	0.0084	0.0051
3	0.0103	0.0099	0.2042	0.0162	0.0090			
4	0.0090	0.0139	0.0454	0.0091	0.0066	0.0906	0.0052	0.0068
5	0.0272	0.0195	0.0100	0.0054	0.0083	0.0368	0.0034	0.0013
6	0.0253	0.0265	0.0302	0.0103	0.0108	0.0518	0.0033	0.0012
7	0.0193	0.0282	0.1932	0.0438	0.0410	0.0178	0.0041	0.0018
8	0.0266	0.0281	0.1130	0.0097	0.0137	0.0867	0.0077	0.0046
9	0.0243	0.0215	0.0187	0.0052	0.0072	0.0283	0.0103	0.0069
10	0.0199	0.0218	0.0467	0.0077	0.0070	0.0416	0.0103	0.0074
11	0.0199	0.0295	0.2178	0.0310	0.0284	0.0102	0.0209	0.0087
12	0.0220	0.0318	0.0700	0.0073	0.0094	0.2198	0.0114	0.0126
13	0.0493	0.0418	0.0097	0.0061	0.0101	0.0311	0.0068	0.0035
14	0.0460	0.0425	0.0292	0.0120	0.0130	0.0440	0.0073	0.0039
15	0.0460	0.0546	0.1655	0.0622	0.0610	0.0227	0.0109	0.0125
16	0.0467	0.0476	0.1406	0.0103	0.0173	0.2147	0.0206	0.0120

MSE1 – Mean Squared Error between the mean simulated values and the predicted values

MSE2 – Mean Squared Error between p0 and the shape values

MSE2_a – Mean Squared Error between p0 and p1

MSE2_b – Mean Squared Error between p1 and p2

The RSF simulations results were fairly consistent across the 16 scenarios with most of the shapes predicting the high predicted survival probability from the method. In this particular analysis, we were able to delineate p0 (baseline), p1 (physician), and p2 (center) levels of learning with the shape being just the actual fitted shape. The number of surgeons did not have much impact but the higher censoring rate of 70% (scenarios 9–16) showed the p1 curve for the logarithmic shape only showed a more typical LC fit. We observed that the MSE for between p0 and p1 for the higher scenarios then tended to be lower as compared to the other models (Table 8).

**Table 8:** – RMST Cox frailty HR per probability levels and scenarios

Scenario	P0	P1	P2
1	1.008	0.942	0.937
2	1.008	1.021	1.058
3			
4	1.008	1.012	1.042
5	1.004	1.088	0.999
6	1.004	1.021	1.044
7	1.004	1.016	1.018
8	1.004	0.989	0.959
9	1.004	1.019	0.997
10	1.004	0.998	1.064
11	1.004	1.049	1.019
12	1.004	1.016	1.006
13	0.988	1.219	1.062
14	0.988	0.955	1.039
15	0.988	1.014	1.042
16	0.988	1.044	0.934

**Fig. 14:**
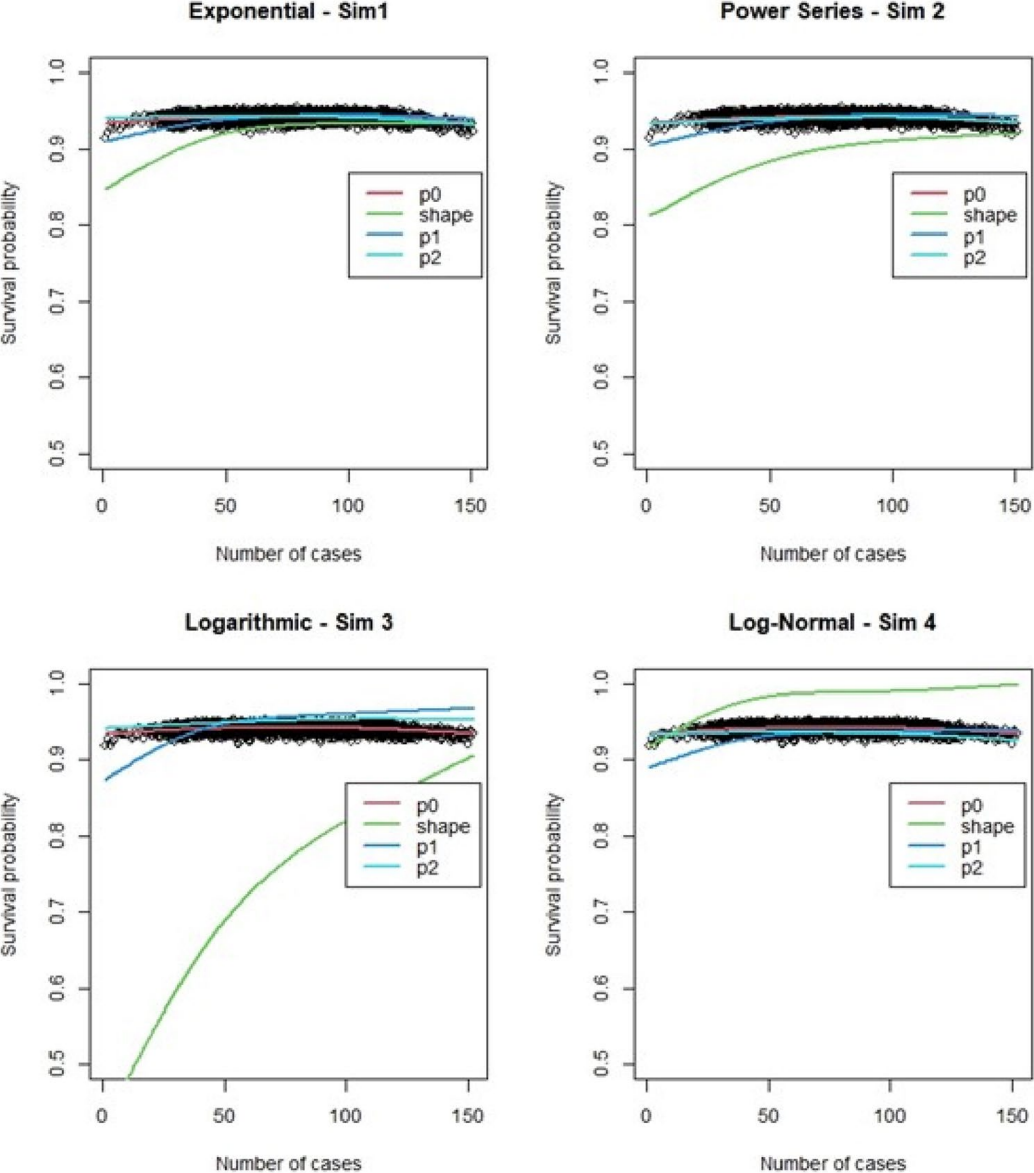
Random survival forests : simulations 1-4 learning curve plots for 4 shapes (exponential, power series, logarithmic, and log-normal) and observed average points

**Fig. 15:**
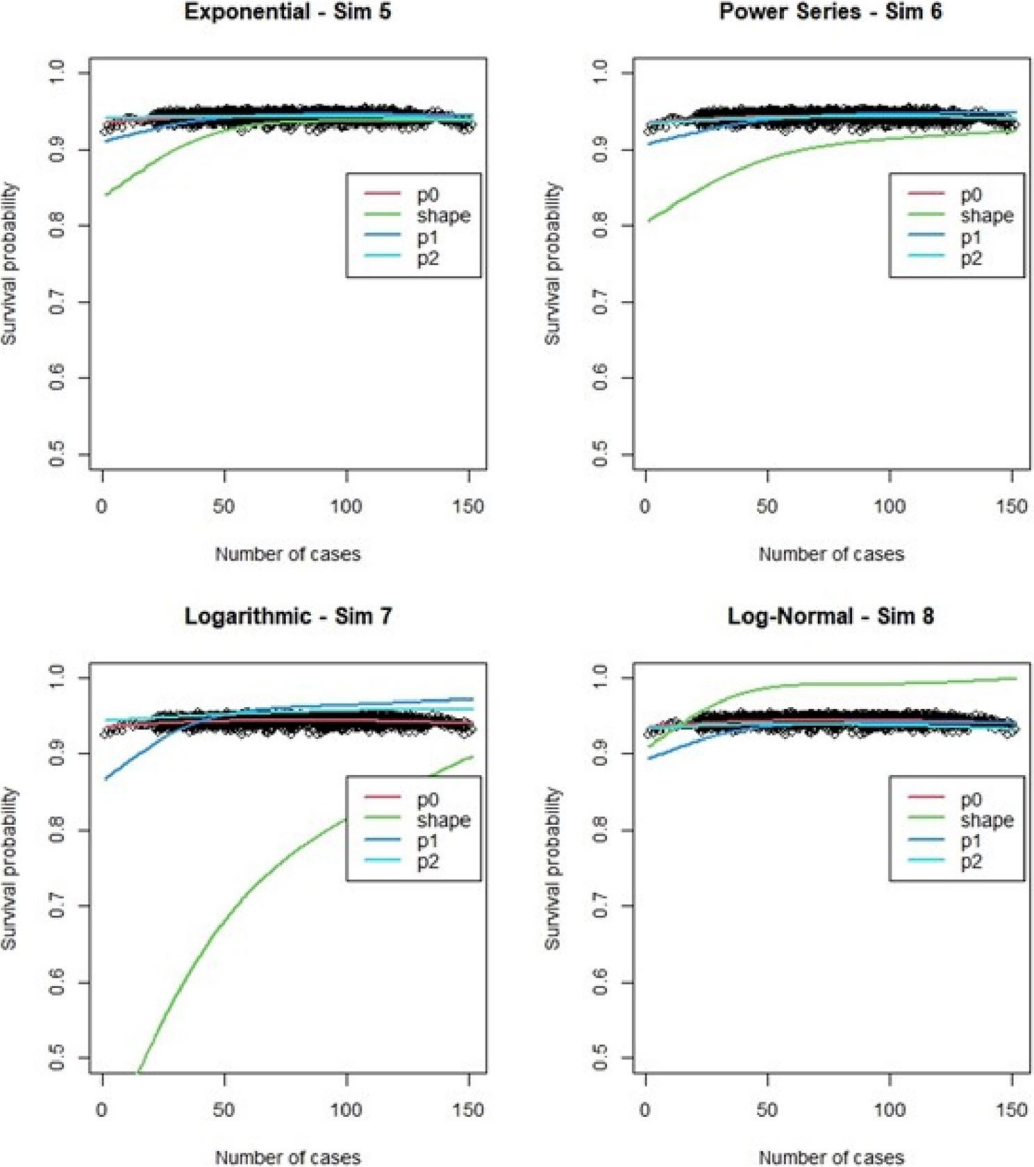
Random survival forests: simulations 5-8 learning curve plots for 4 shapes (exponential, power series, logarithmic, and log-normal) and observed average points

**Fig. 16:**
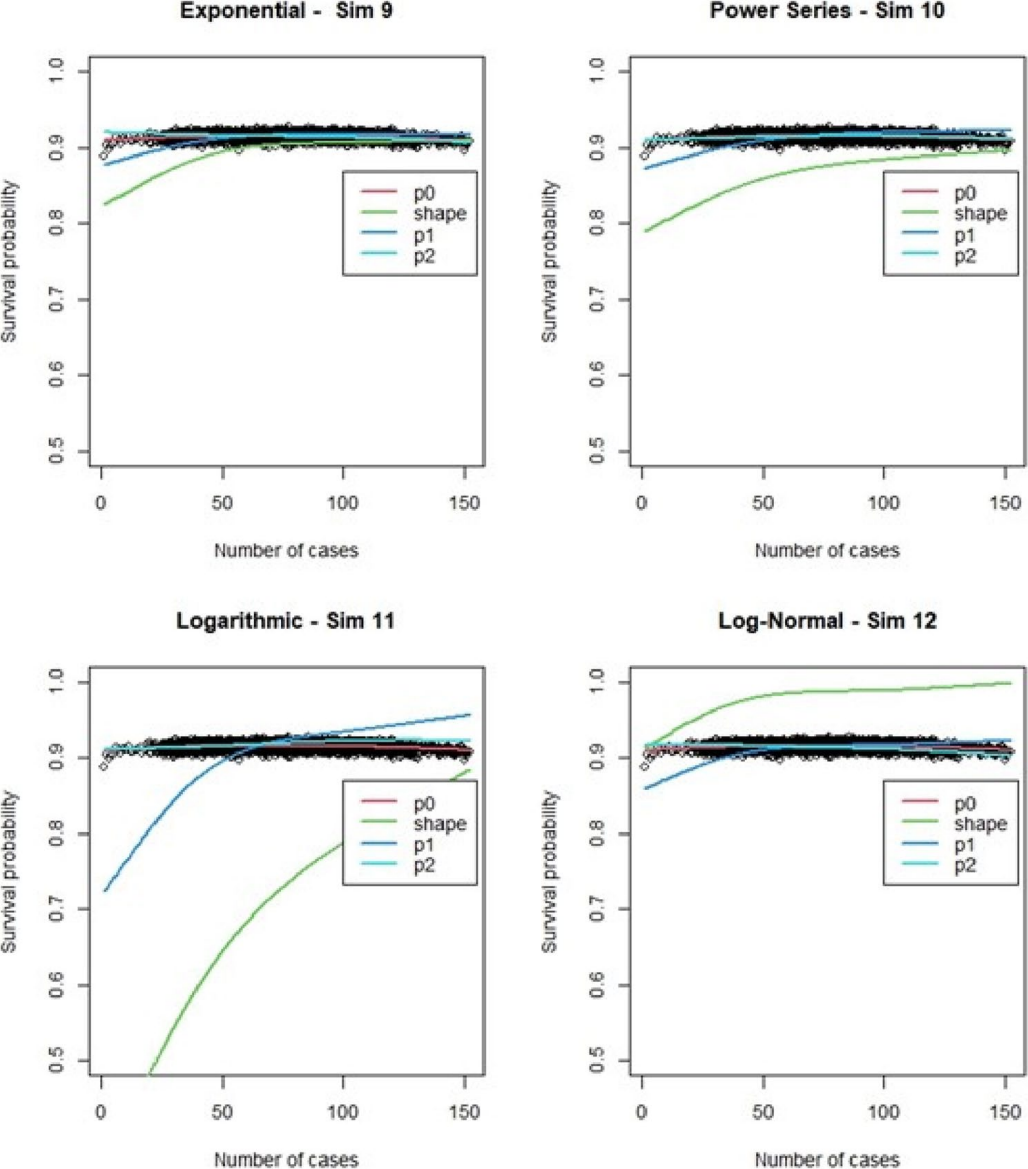
Random survival forests: simulations 9-12 learning curve plots for 4 shapes (exponential, power series, logarithmic, and log-normal) and observed average points

**Fig. 17:**
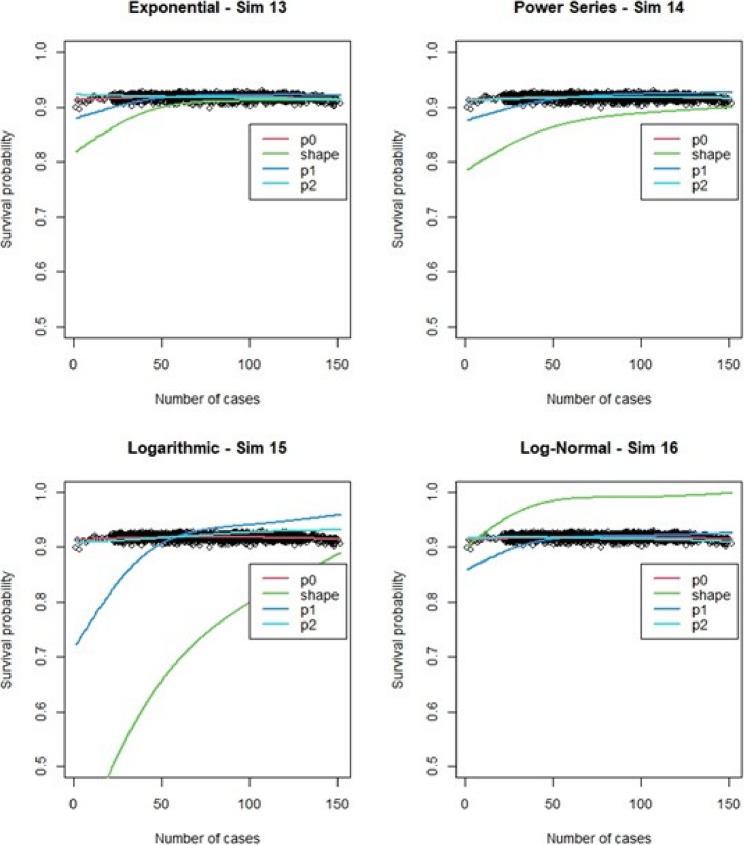
Random survival forests: simulations 13-16 learning curve plots for 4 shapes (exponential, power series, logarithmic, and log-normal) and observed average points

For the Cox RMST frailty model, unlike the other models since we did not obtain individual predicted survival from this modeling, we only collected the MSE and hazard rates for each scenario. We also ran into an issue with scenario 3 only where RAM issues kept causing the software to crash. Across the scenarios the logarithmic shape function performed best. Interestingly both the logarithmic model and the log-normal model increased the observable MSE between the default model and the model adjusted for surgeons, as well as the MSE between the surgeon adjusted model and the surgeon & site adjusted model. This suggested that although the logarithmic performed better overall, it did suppress some of the effects of surgeon and sites in the model (Figs. 14,15, 16, and 17).

## Discussion

From the main survival analysis results presented and as per prior analyses [1], we saw that age was the main predictor in post-procedure time to death analysis. Other variables appeared to have had significant effects in the simple model that were not present in the adjusted model. This indicated the possible presence of an unobserved confounding variable or variables that had significant effects on the mortality outcome. We demonstrated that besides standard survival analysis which was important to assess for these EVD procedures that also employing more unique analyses with modeling unexplained heterogeneity through frailty model could lead to other results. The Cox frailty model as well as our RMST Cox frailty model showed that smoking status was the main predictor of mortality in the presence of any latent heterogeneity within the model, even though the frailty effect itself was not statistically significant in either model. This suggested that incorporating modeling frailty may have shown a more genuine causal effect between a predictor of mortality amongst these stroke patients who had an EVD procedure.

In the time to complication analysis the type of EVD device showed a statistically significant effect, with Bactericidal devices performing better than IRRAflow/Cerebroflo© in terms of post-operative complications times. Also, pre-operative Modified Rankin Score showed a significant effect in univariate analysis and a borderline effect in the multivariate analysis, aligning with expectations of it to be a significant predictor. Meanwhile age and smoking did not appear to show significant effects in these analyses.

In the real data analysis of the learning curve, we observed a common trend across all plotted models, a brief decrease in probability of success. The main observable differences between models was were the timing and severity of this dip. For the Cox model analyses, a substantial dip occurred after 40 procedural cases while for the Cox frailty model analyses, the dip was less severe and occurred around 20 procedural cases. Finally for the RSF, only very minor dips were observed around 30 procedural cases. We believe this observed temporary dip was likely the result of mentorship. Often a more experienced practitioner would supervise a surgeon until they were more familiar with the procedure. This would have resulted in higher initial probability of success while the mentor was providing assistance. We posit that removing this mentoring effect might reveal a more logarithmic learning curve, characterized by low initial probability of success that increases over time. This hypothesis is supported by the observed behavior in our simulated models.

The simulated learning curves demonstrated consideration variation across the models and scenarios, yet some consistent trends could be observed. The logarithmic shape function generally performed similar to or better than the other shape functions, aligning with logical expectations of high learning rates that plateau as the model approaches an asymptotic limit. The primary differences between models were evident in their sensitivity to varying parameters across simulations. The Cox frailty model showed great differences as the censoring rate and number of surgeons were varied but it was not as affected by changes in the shape as compared to the regular Cox model. The intercept of the regular Cox model tended to vary significantly according to adapting the rate of censoring and the accuracy of the predictions from this model appeared to vary according to the number of surgeons per scenario. The use of different shapes for the LC appeared to influence the slope of the predicted values. In regard to the RSF, the method appeared desensitized to the parameter values and this was observed across all scenarios.

In comparing the simulated results and the real data analysis, it appeared that one of the most significant factors that affected learning rates may have been the presence of a mentor or supervisor. Further research should be performed to investigate if this observed effect is the result of mentorship, how this effect varies across mentors, how do we determine the actual learning rate when surgical outcomes are being adjusted by supervisors and how do we optimize learning rate while minimizing negative surgical outcomes through the application of mentorship.

## Data Availability

Due to the proprietorial nature of the data source, it will not be made publicly available.
